# Identifying Depression Through Machine Learning Analysis of Omics Data: Scoping Review

**DOI:** 10.2196/54810

**Published:** 2024-07-19

**Authors:** Brittany Taylor, Mollie Hobensack, Stephanie Niño de Rivera, Yihong Zhao, Ruth Masterson Creber, Kenrick Cato

**Affiliations:** 1 School of Nursing Columbia University New York, NY United States; 2 Brookdale Department of Geriatrics and Palliative Care Icahn School of Medicine Mount Sinai Health System New York, NY United States; 3 School of Nursing University of Pennsylvania Philadelphia, PA United States

**Keywords:** machine learning, depression, omics, review, mental health, nurses

## Abstract

**Background:**

Depression is one of the most common mental disorders that affects >300 million people worldwide. There is a shortage of providers trained in the provision of mental health care, and the nursing workforce is essential in filling this gap. The diagnosis of depression relies heavily on self-reported symptoms and clinical interviews, which are subject to implicit biases. The omics methods, including genomics, transcriptomics, epigenomics, and microbiomics, are novel methods for identifying the biological underpinnings of depression. Machine learning is used to analyze genomic data that includes large, heterogeneous, and multidimensional data sets.

**Objective:**

This scoping review aims to review the existing literature on machine learning methods for omics data analysis to identify individuals with depression, with the goal of providing insight into alternative objective and driven insights into the diagnostic process for depression.

**Methods:**

This scoping review was reported following the PRISMA-ScR (Preferred Reporting Items for Systematic Reviews and Meta-Analyses Extension for Scoping Reviews) guidelines. Searches were conducted in 3 databases to identify relevant publications. A total of 3 independent researchers performed screening, and discrepancies were resolved by consensus. Critical appraisal was performed using the Joanna Briggs Institute Critical Appraisal Checklist for Analytical Cross-Sectional Studies.

**Results:**

The screening process identified 15 relevant papers. The omics methods included genomics, transcriptomics, epigenomics, multiomics, and microbiomics, and machine learning methods included random forest, support vector machine, k-nearest neighbor, and artificial neural network.

**Conclusions:**

The findings of this scoping review indicate that the omics methods had similar performance in identifying omics variants associated with depression. All machine learning methods performed well based on their performance metrics. When variants in omics data are associated with an increased risk of depression, the important next step is for clinicians, especially nurses, to assess individuals for symptoms of depression and provide a diagnosis and any necessary treatment.

## Introduction

### Significance of Depression

Depression is one of the most common mood disorders, with a prevalence of approximately 20% in adults in the United States [1,2]. Among people with diagnosed depression, nearly half experience severe depression, and 40% experience moderate depression [1]. Between 2010 and 2018, the number of adults in the United States diagnosed with depression increased by 13%, and the associated health care costs also increased, including medical and pharmaceutical costs, workplace absenteeism, and suicide-related costs [3]. Despite a greater investment in mental health, approximately half of the people experiencing depression have been diagnosed and treated [4]. There have been limited improvements in the mental health care of depression during the past decade, primarily owing to the challenges in accurately diagnosing this complex illness [5]. Consequently, there is an urgent imperative to explore and establish more objective diagnostic approaches that can better identify individuals with depression and pave the way for more effective interventions and personalized treatment strategies.

### Diagnostic Methods for Depression

The gold standard for depression diagnosis involves a structured psychiatric interview [2] that includes validated depression scales such as the Center for Epidemiologic Studies–Depression Scale, Hamilton Rating Scale for Depression-17, Montgomery-Asberg Depression Rating Scale, and the Beck Depression Inventory [6]. While these validated scales can be administered by a trained interviewer, a licensed mental health provider is required to make a formal diagnosis [2]. This method, while routinely used, is subjective to the clinician conducting the interview, leading to potential variations in diagnosis.

There are several other barriers to the diagnosis of depression, which include limited access to health care services and societal stigma toward mental health diagnoses. The *Diagnostic and Statistical Manual of Mental Disorders* defines depression as a heterogenous disorder that is diagnosed based on the core symptoms of depressed mood or anhedonia and at least 4 of the 9 other symptoms, including appetite changes, sleep changes, fatigue, difficulty in concentrating, feeling worthless, and suicidal ideation; depression is present if these symptoms last for at least 2 weeks [5]. Furthermore, the heterogeneity of symptoms in depression makes diagnosis difficult [7], and it is described differently across cultures [8]. In addition, there is social stigma and perceived conflict with normative social roles that prevent many patients from being honest about their thoughts and feelings [6].

### Nursing Care for Depression

Second to social work, nursing is the largest profession in the mental health workforce [9]. In 2013, it was estimated that 4% of the total registered nursing workforce provided mental health care, and in 2015, the number was estimated by the National Nursing Workforce Survey to be 134,000 registered nurses [9]. Advanced practice registered nurses are a vital part of the mental health workforce, especially in rural areas where there are few licensed mental health professionals with prescribing capabilities [9].

### Genomics of Depression

Owing to multilevel biases around diagnoses of depression, including implicit bias of providers, social desirability bias of patients, and bias introduced by data processing, alternative methods for an objective biologically informed diagnosis are being explored [10,11]. Currently, biomarkers, such as single nucleotide polymorphisms (SNPs), messenger RNA (mRNA), microRNA, proteins, and methylated DNA, are being sequenced and combined with scores on standardized depression instruments to evaluate whether they can improve the sensitivity and specificity of a depression diagnosis. Ideally, biomarker profiling would be performed on brain tissue, as it offers valuable insights into the underlying neurobiological mechanisms [6]. However, brain biopsies are dangerously invasive, so peripheral blood or saliva is often used as an alternative sample type [6]. Importantly, recent studies have shown a high correlation in gene expression and methylation patterns between blood and saliva samples and brain tissue, supporting the utility of peripheral samples as valuable surrogates for understanding the molecular mechanisms underlying depression [12-14]. Therefore, this study focuses on studies that use blood or saliva sample types for the diagnosis of depression.

The heritability of depression is estimated to be 40%, and many studies have been performed to identify genetic variants or SNPs that are associated with depression [15,16]. Genomic analysis can be performed through genome-wide association studies (GWASs). The 2 types of GWAS are classical and functional. Classical GWAS identifies SNPs that are associated with specific traits or diseases [15]. Functional GWAS determines how SNPs overlap with regulatory elements such as enhancers and promotors and predicts how these SNPs function [15]. A GWAS of samples in the Taiwan Biobank identified SNPs in 17 different genes that were significantly associated with depression [16]. Results from GWAS analyses suggest that depression is a polygenic disorder, meaning many SNPs can affect the hereditary influence [4]. SNPs identified through GWASs can be used to compute polygenic risk scores [4]. Polygenic risk scores combine the effects of genetic variants into an overall score that reflects an individual’s propensity for a disease [17].

### Transcriptomics of Depression

The transcriptome is all of the body’s mRNA and contains coding instructions for protein synthesis [18,19]. Transcriptome analysis is useful for measuring gene expression. Recently developed sequencing techniques allow the expression levels of thousands of transcripts to be measured simultaneously [19]. Differentially expressed genes (DEGs) in patients with depression and healthy controls have been identified in both peripheral blood samples and brain tissues [18].

### Epigenomics of Depression

Epigenetics leads to heritable changes in gene expression without affecting the underlying genetic sequences [20]. Studies have shown that epigenetics may be as influential as genetic variants in the development of depression [21]. Two types of epigenetic modifiers are DNA methylation (DNAm) and microRNA. DNAm occurs at sites in the genetic sequence where the nucleotides cytosine and guanine are bound together in clusters known as cytosine-phosphodiester bond-guanine (CpG) islands [21]. DNAm is responsive to environmental stimuli and can affect gene expression by inhibiting the transcription of affected genes [21]. MicroRNAs are small, noncoding RNAs up to 25 nucleotides in length [20]. Unlike mRNA, they are not translated into protein. Instead, they bind to mRNA to suppress protein translation, leading to decreased gene expression [20]. The effects of several microRNAs have been found to be upregulated or downregulated in individuals with depression [1].

In some studies, >1 sequencing method is used on the samples to produce different types of omics data. In the multiomics study by Bhak et al [6], blood samples were sequenced using Methyl-Seq to produce epigenomic data and RNA-Seq to produce transcriptomic data. Using these data, the authors were able to distinguish between people with depression who have attempted suicide, people with depression who have not attempted suicide, and healthy controls [6]. Combining >1 omics data type can improve prediction accuracy [6].

### Microbiomics of Depression

The diversity of microbiota in the gut is influenced by genetics, development, and environment [22]. In the gut microbiome, the gut microbiota transmit signals to the brain through pathways associated with neural transmission and control of behaviors [22]. Depression has been associated with gut dysbiosis, an imbalance of the gut microbiota that is associated with adverse health outcomes [23,24]. Some strains of bacteria have been associated with depression in multiple studies, including *Eggerthella*, *Subdoligranulum*, *Coprococcus*, and *Ruminococcaceae* [25]. Furthermore, studies have found differences in metabolic pathways between individuals with depression and healthy controls [24].

### Machine Learning Methods to Identify Individuals With Depression From Omics Data

Omics data are inherently complex and often too large for manual evaluation [26]. Machine learning, a form of artificial intelligence, is useful for detecting subtle patterns in large data sets, allowing it to predict multifactorial diseases [11,27]. By training algorithms on data, machine learning models identify patterns and make predictions that may be beyond human capabilities [28]. Machine learning algorithms can be supervised, where the algorithm learns from labeled training data to make predictions in unlabeled testing data, or unsupervised, where there is no labeling, and the algorithm categorizes the data into groups or finds complex patterns [29].

Machine learning models are being investigated to aid in the development of predictive algorithms to help understand how genetic variation can affect disease status [16]. A key aspect of machine learning is feature selection, which helps determine the importance of each feature and its contribution to the model’s performance during training; in omics data, features can encompass various entities, such as SNPs, DEGs, or DNAm sites [6]. Machine learning can be useful for analyzing transcriptomic data because traditional statistical methods may not fully capture molecular interactions between genes [30].

Through machine learning, researchers can not only identify genes associated with a specific disease but also explore linear and nonlinear gene interactions [30]. While there is great potential in using machine learning to advance omics knowledge on depression, no prior studies have summarized the machine learning methods used to analyze omics data for depression. Therefore, this scoping review aims to provide an overview of the existing literature on using machine learning methods to analyze omics data to identify individuals with depression.

## Methods

This scoping review was reported following the PRISMA-ScR (Preferred Reporting Items for Systematic Reviews and Meta-Analyses Extension for Scoping Reviews) guidelines [31].

### Search Strategies

Searches were conducted in 3 databases between November and December 2022: PubMed, CINAHL, and Scopus. The search strategy used terms representing machine learning; depression; and different types of omics, including genomics, transcriptomics, and epigenomics (Multimedia Appendix 1). Keywords were combined using Boolean operators.

### Selection Criteria

After deduplication, 3 independent reviewers (BT, MH, and SN) conducted pairwise screening of titles and abstracts with specific inclusion and exclusion criteria using Covidence (Veritas Health Innovation) systematic review web software. This resulted in a set of papers for full-text review that were also reviewed pairwise, with disagreements resolved by consensus. Specific inclusion criteria consisted of studies published in peer-reviewed journals, English, and the past 5 years (ie, between January 1, 2017, and December 31, 2022). Publication dates were limited to the past 5 years because genetic sequencing is constantly evolving, and older studies may have used outdated methods [32]. Furthermore, all studies had to include (1) an omics method involving the sequencing of genetic material to identify depression and (2) an approach that used machine learning or deep learning to analyze the omics data. Papers were excluded if they focused on omics methods that did not involve sequencing of genetic material, such as metabolomics and lipidomics. In addition, review papers; deep learning studies of medical images; and studies focusing on other disorders, such as bipolar disorder, anxiety disorder, posttraumatic stress disorder, and schizophrenia, were excluded.

Any disagreements between screeners were discussed and resolved through consensus. After the initial screening, full texts of the remaining papers were reviewed. Reference lists were also screened to identify any additional papers meeting the inclusion criteria. Covidence software was used throughout the screening process. Data charting was completed for the eligible studies using Word (Microsoft Corp).

### Data Extraction

Items extracted included author, year, study design, and sample size. Data extracted included the omics type, machine learning method, sample type, and depression screening instrument used. Charted data were synthesized by grouping studies according to their omics method (eg, genomics and transcriptomics).

Critical appraisal was performed using the Joanna Briggs Institute Critical Appraisal Checklist for Analytical Cross-Sectional Studies [33]. This checklist was chosen because the genomic data in the studies included in this review were analyzed at a single point in time [34]. The checklist appraises inclusion criteria, measurement of exposure and outcomes, confounding, and statistical analysis. Questions are answered as yes, no, unclear, or not applicable [33].

## Results

### Search Summary

**Figure 1:**
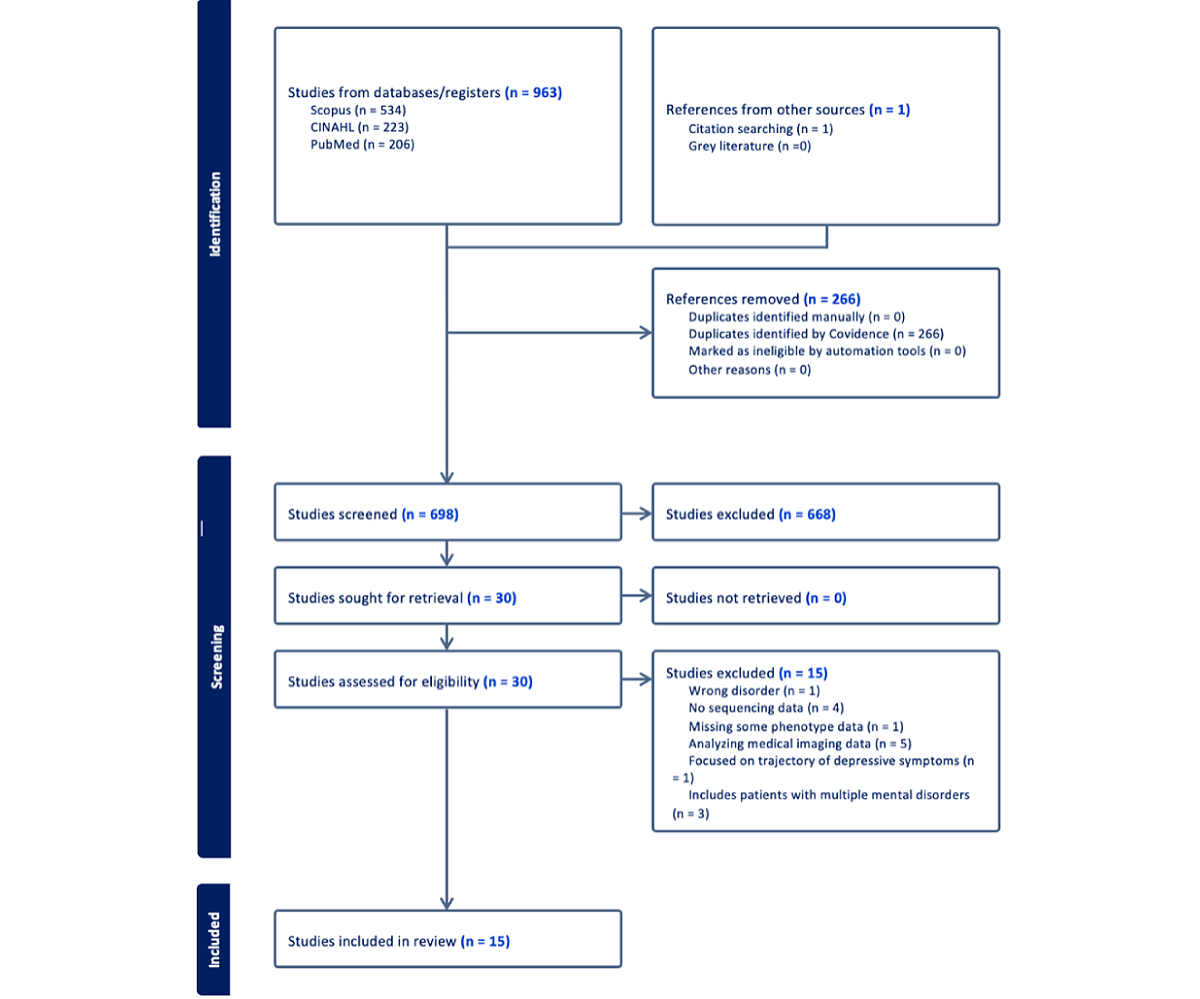
PRISMA (Preferred Reporting Items for Systematic Reviews and Meta-Analyses) flow diagram.

The initial database search yielded 964 papers; 266 (27.6%) papers were removed as duplicates. Of the 964 papers, the titles and abstracts of 698 (72.4%) papers were screened for eligibility. A priori exclusion criteria were applied throughout the title and abstract screening of the 698 papers, and 668 (95.7%) papers were excluded. Of the 698 papers, 30 (4.3%) met the criteria for full-text review and were assessed for eligibility, of which 15 (50%) were included in this scoping review. This screening process is visualized in a PRISMA (Preferred Reporting Items for Systematic Reviews and Meta-Analyses) flow diagram (Figure 1).

### Summary of Study Characteristics

The included studies were published between 2017 and 2022. The studies were conducted in 8 countries: Germany (1/15, 7%), South Korea (1/15, 7%), Australia (1/15, 7%), China (1/15, 7%), Taiwan (1/15, 7%), Canada (2/15, 13%), United States (6/15, 40%), Japan (1/15, 7%), and India (1/15, 7%). All the studies were cross-sectional design studies. The studies addressed genomics (5/15, 33%), transcriptomics (5/15, 33%), epigenomics (3/15, 20%), multiomics (1/15, 7%), and microbiomics (1/15, 7%). Machine learning methods included random forest, support vector machine, k-nearest neighbor, artificial neural network, and deep learning. Study characteristics are further described in Table 1.

**Table 1:** Study characteristics.

Type of omics and study		Country	Sample size, n	Age range	Depression diagnosis	Screening instrument
**Genomics**						
	Arabnejad et al [35], 2018	United States	922 (463 cases and 459 controls)	Not given	Screening	Composite International Diagnostic Interview–Short Form Structured Clinical Interview for DSM-IV^a^ Patient Health Questionnaire-9
	Arloth et al [15], 2020	Germany	3514 (1476 cases and 2038 controls)	Not given	Not given	Not given
	Lin et al [16], 2021	Taiwan	9828 (2457 cases and 7371 controls)	Mean 51.2 (SD 10.4) years	Psychiatrist	Patient Health Questionnaire
	Sekaran and Sudha [26], 2019	United States	100 (66 cases and 34 controls)	Not given	Not given	Not given
	Takahashi et al [36], 2020	Japan	6733 (185 cases and 6548 controls)	Mean 60 (SD 11) years	Not given	Center for Epidemiological Studies–Depression Scale
**Transcriptomics**						
	Ciobanu et al [30], 2020	Australia	521 (27 cases and 494 controls)	70 to 90 years	Screening	Geriatric Depression Scale Patient Health Questionnaire Neuropsychiatric Inventory
	Le et al [37], 2020	United States	157 (78 cases and 79 controls)	Not given	Psychiatrist	Montgomery-Asberg Depression Rating Scale
	Parvandeh et al [38], 2020	United States	915 (463 cases and 452 controls)	Not given	Screening	Composite International Diagnostic Interview–Short Form Structured Clinical Interview for DSM-IV Patient Health Questionnaire-9
	Qi et al [18], 2021	Canada	2295 (1765 cases and 530 controls)	>18 years	Not given	Not given
	Verma and Shakya [19], 2022	India	59 (30 cases and 29 controls)	Not given	Not given	Not given
**Epigenomics**						
	Fan et al [27], 2021	China	391 (291 cases and 100 controls)	18 to 65 years	Psychiatrist	Hamilton Rating Scale for Depression-17
	Payne et al [39], 2020	United States	267 (54 cases and 213 controls)	Not given	Screening	Edinburgh Postnatal Depression Scale
	Qi et al [1], 2020	Canada	168 (140 cases and 28 controls)	Not given	Psychiatrist	Montgomery-Asberg Depression Rating Scale
**Microbiomics**						
	Stevens et al [24], 2021	United States	40 (20 cases and 20 controls)	Not given	Psychiatrist	None
**Multiomics**						
	Bhak et al [6], 2019	South Korea	182 (95 cases and 87 controls)	19 to 46 years	Psychiatrist	Hamilton Rating Scale for Depression-17

^a^DSM-IV: Diagnostic and Statistical Manual of Mental Disorders (Fourth Edition).

### Genomics

One study combined classical and functional GWASs and annotated SNPs based on their regulatory potential and combination with a functional unit (FU) [15]. This method is called a multivariate FU-wide association study (DeepWAS) [15]. A DeepWAS can identify SNPs associated with a disease (dSNPs) [15]. A DeepWAS successfully identified 61 dSNPs in 237 FUs that were associated with depression; 60 (25.3%) of these dSNPs were significant (Table 2) [15]. To validate these results, the dSNPs were compared to SNPs identified by other GWASs [15]. A total of 4 dSNPs overlapped with a large GWAS by the UK Biobank: the *LARP6-LRRC*49 gene, 2 intergenic regions near the *WNT2* and *ASZ1* genes, the *ATG9B* and *ABCB8* genes on chromosome 7, and a site near the *C1orf220* and *MIR4424* genes on chromosome 1 [15]. In addition, the DeepWAS identified an SNP on the transcription factor binding site of *MEF2C* on chromosome 8 as a regulator for depression [15]. The GWAS using data collected from 2 prefectures in Japan included 102 SNPs in the model with the highest prediction accuracy [36]. However, none of these variants were significant at the 5.0×10^–8^ level, and the top 11 variants only explained 0.0036% of the variance in the validation data set, which is a very small effect size [36].

Using data from the Taiwan Biobank, a novel SNP, rs192922209, located in the intron region of the *FBN1* gene on chromosome 15, was associated with depression [16]. In addition, a novel SNP was associated with depression in female individuals: rs114542799 in the intron region of the *ALDH1L1* gene on chromosome 3 [16]. Furthermore, this study identified 17 SNPs with potential roles as expression quantitative trait loci [16]. Arabnejad et al [35] used GWAS data to identify significant SNPs and their associated genes to test for pathways that overlap with depression. They identified the top 500 SNPs using different feature selection methods and compared the number of genes detected to the biological pathways [35]. Pathways that previous studies have associated with depression were reported: axon guidance pathway, neuronal system pathway, and pathways related to G protein–coupled receptors, which affect neurotransmitter signaling [35].

Sekaran and Sudha [26] aimed to identify genetic variants related to depression by using DNA microarrays. Sample participants were classified into 3 categories: patients with depression with lipopolysaccharide treatment, patients with depression without lipopolysaccharide treatment, and healthy controls [26]. A total of 27 genetic biomarkers associated with depression were identified; the biomarker *A_23_P109436*, was able to classify the data with the highest precision [26].

**Table 2:** Study findings.

Type of omics and study			Sample type		Key findings
**Genomics**					
	Arabnejad et al [35], 2018	Blood		Detected pathways associated with depression, including axon guidance, neuronal system, and G protein–coupled receptor signaling	
	Arloth et al [15], 2020	Not given		Identified 61 dSNPs^a^ in 237 FUs^b^; 60 of the dSNPs were significant A total of 4 dSNPs were also found in a GWAS^c^ by the UK Biobank A SNP^d^ on the MEF2C gene was identified as a regulator for depression	
	Lin et al [16], 2021	Blood		This study identified a novel SNP on the FBN1 gene associated with depression A novel SNP on the ALDH1L1 was associated with depression in female individuals A total of 17 SNPs with potential roles as expression quantitative trait loci were pinpointed	
	Sekaran and Sudha [26], 2019	Not given		Identified 27 genetic biomarkers associated with depression A biomarker, A_23_P109436, classified the data with the highest precision	
	Takahashi et al [36], 2020	Blood		The model with the highest prediction accuracy included 102 SNPs None of these SNPs were significant at the 5.0×10–8 level	
**Transcriptomics**					
	Ciobanu et al [30], 2020	Blood		Downregulation of the transferrin receptor gene is associated with depression	
	Le et al [37], 2020	Blood		Identified 23 depression gene modules	
	Parvandeh et al [38], 2020	Blood		The best performing model had a significant overlap of 959 genes with the initial 7616 genes (*P*<.001)	
	Qi et al [18], 2021	Brain and blood		Analysis of brain mRNA^e^ revealed 62 DEGs^f^ used to distinguish cases from controls Analysis of blood mRNA found 1376 DEGs	
	Verma and Shakya [19], 2022	Blood		A total of 624 transcripts correlated with the classification of patients with depression who died by suicide, those who did not die by suicide, and healthy controls	
**Epigenomics**					
	Fan et al [27], 2021	Blood		Identified 9 differentially methylated sites on the tryptophan hydroxylase-2 gene	
	Payne et al [39], 2020	Blood		Found that DNAm^g^ in the first trimester could accurately predict depression in the third trimester Third-trimester DNAm predicted postpartum depression	
	Qi et al [1], 2020	Blood		A total of 4 microRNAs differed significantly, but these differences were not significant	
**Microbiomics**					
	Stevens et al [24], 2021	Stool		Found decreased amounts of Faecalibacterium, Ruminococcus, Lachnospiraceae, and Bacterioides species in the microbiomes of the individuals in the group with depressive symptoms	
**Multiomics**					
	Bhak et al [6], 2019	Blood		Identified 48 DEGs and 810 differentially methylated sites that significantly correlated with depression scores	

^a^dSNPs: single nucleotide polymorphisms associated with a disease.

^b^FU: functional unit.

^c^GWAS: genome-wide association study.

^d^SNP: single nucleotide polymorphism.

^e^mRNA: messenger RNA.

^f^DEG: differentially expressed gene.

^g^DNAm: DNA methylation.

### Transcriptomics

Ciobanu et al [30] used transcriptomic data to identify a link between depression and the transferrin receptor gene on chromosome 3. When downregulated, this gene is associated with recurrent depression [30]. In the study by Verma and Shakya [19], differential gene expression was examined between patients with depression who died by suicide, those who did not die by suicide, and healthy controls. A total of 624 transcripts were found to be biologically and functionally related to classifying the 3 categories [19]. Most of these transcripts were associated with neurotransmitter receptors, postsynaptic signal transmission, synaptic depression, gamma-aminobutyric acid receptor activation, and glutamatergic synapse [19].

Using RNA sequence data, Parvandeh et al [38] aimed to classify patients with depression and healthy controls. They analyzed 7616 genes that are known to be associated with depression based on prior studies; these genes were compared to a repository of genes associated with mental disorders from the DisGeNET platform [38]. The best performing model had an overlap of 959 genes with the initial 7616 genes and *P*<.001, indicating significant overlap [38]. Using brain mRNA to discriminate between cases and controls, the best performing model identified 62 DEGs [18]. These genes were associated with upregulation of metalloaminopeptidase activity, downregulation of oxidoreductase activity, and upregulation of aminopeptidase activity [18]. Furthermore, this study used blood mRNA to identify 1376 DEGs associated with depression [18]. RNA-Seq Rdata was used to identify depression gene modules (DGMs), genes that are interconnected and coexpressed, and predict a clinical diagnosis of depression [37]. A total of 23 DGMs were identified; DGM-5 was most predictive of depression diagnosis and was significantly associated with depression severity [37].

### Epigenomics

In the epigenetic study of postpartum depression by Payne et al [39], the authors used DNAm biomarker profiles on the *TTC9B* and *HP1BP3* genes to predict antenatal and postpartum depression [39]. A total of 4 separate cohorts were included in this study, and blood samples were drawn during different trimesters of pregnancy [39]. They found that DNAm biomarkers from samples collected during the first trimester could accurately predict depression in the third trimester [39]. In addition, biomarker profiles in third-trimester samples predicted depression in the postpartum period [39].

The DNAm study by Fan et al [27] focused on methylation of the tryptophan hydroxylase-2 gene, which functions in the production of serotonin. They identified 9 CpG sites on the tryptophan hydroxylase-2 gene that differ significantly between patients with depression and healthy controls [27]. In the microRNA study by Qi et al [1], 4 microRNAs were found to differ significantly between patients with depression and healthy controls. However, none of these microRNAs remained significant after Bonferroni correction [1].

### Microbiomics

One study used genomic variants in the microbiome to distinguish between individuals with depression and healthy controls [24]. After examining exact amplicon sequence variants, biological sequences that have been inferred through shotgun sequencing, the authors found decreased abundances of *Faecalibacterium*, *Ruminococcus*, *Lachnospiraceae*, and *Bacterioides* species in the microbiomes of the individuals in the depression group compared to those in the healthy group [24]. Furthermore, they found that pathways involved in the degradation of the neurotransmitter gamma-aminobutyric acid and the fatty acid butyrate were more prominent in individuals with depression [24].

### Multiomics

The multiomics study using blood transcriptome and methylome data identified DEGs and differentially methylated sites (DMSs) in individuals with depression and controls [6]. This study included 3 cohorts: 56 individuals with depression who attempted suicide, 39 individuals with depression who did not attempt suicide, and 87 healthy controls [6]. A total of 80 DMSs were identified between individuals with depression who did not attempt suicide, and 95 DMSs and 7 DEGs were identified between individuals with depression who attempted suicide and controls [6]. Between individuals with depression who did and did not attempt suicide, 69 DMSs were found [6]. In addition, 48 DEGs and 810 DMSs were significantly correlated with scores on the Hamilton Rating Scale for Depression-17 [6]. A functional enrichment test was conducted to investigate pathways associated with the model input features. A difference in enrichment was detected between depressed individuals who did not attempt suicide “and controls in the Hippo signaling pathway, which includes the Protein Kinase C gene on chromosome 2 and the Frizzled Class Receptor 7 gene on chromosome 1 [6]. In addition, protocadherin genes were enriched in depressed individuals who attempted suicide compared to controls [6].

### Supervised Machine Learning

In an epigenomic study, linear discriminant analysis and support vector machine were used to predict depression in the first, second, or third trimester of pregnancy [39]. Linear discriminant analysis predicted depression in the third trimester with an accuracy >70% and an area under the curve (AUC) of 0.72 (Table 3); similarly, support vector machine predictions for the same trimester had an accuracy of 72% and AUC of 0.83 [39]. Support vector machine also successfully identified women with depression in the postpartum period with an AUC of 0.78; an AUC >0.5 indicates the model has some level of discriminatory ability and can adequately distinguish between cases and controls better than random chance [39].

**Table 3:** Machine learning methods and performance metrics.

Type of omics, study, and machine learning method				AUC^a^		Accuracy		Sensitivity		Specificity
**Genomics**										
	**Arabnejad et al [35], 2018^b^**									
		ReliefF	—^c^		—		—		—	
		Random forest	—		—		—		—	
		Lasso regression	—		—		—		—	
	**Arloth et al [15], 2020**									
		DeepWAS^d^ or DeepSEA^e^	0.59-0.66		—		—		—	
	**Lin et al [16], 2021**									
		Random forest	0.82		—		0.76		0.76	
		Support vector machine	0.76		—		0.76		0.76	
		Decision tree	0.76		—		0.76		0.76	
		Logistic ridge regression	0.82		—		0.76		0.76	
		LogitBoost	0.82		—		0.76		0.76	
	**Sekaran and Sudha [26], 2019**									
		Bayesian network	—		*0.96^f^*		—		—	
		Support vector machine	—		0.73		—		—	
		Random forest	—		0.91		—		—	
		Neural network	—		0.72		—		—	
		Linear discriminant analysis	—		0.70		—		—	
	**Takahashi et al [36], 2020^g^**									
		Smooth-threshold multivariate genetic prediction	—		—		—		—	
		Genomics best linear unbiased prediction	—		—		—		—	
		Summary data–based best linear unbiased prediction	—		—		—		—	
		Bayes regression	—		—		—		—	
		Ridge regression	—		—		—		—	
**Transcriptomics**										
	**Ciobanu et al [30], 2020**									
		Fuzzy forest	—		0.63		0.63		0.66	
	**Le et al [37], 2020**									
		Tree-based pipeline optimization tool	—		*0.48-0.65*		—		—	
		Extreme gradient boost	—		0.49-0.59		—		—	
	**Parvandeh et al [38], 2020**									
		Consensus nested cross-validation	—		*0.59*		—		—	
		Nested cross-validation	—		0.56		—		—	
		Private evaporative cooling	—		0.58		—		—	
		General Elastic net	—		0.51		—		—	
	**Qi et al [18], 2021**									
		Extreme gradient boost	0.55-0.72		0.67-0.85		—		—	
		Logistic regression	*0.62-0.91*		—		—		—	
	**Verma and Shakya [19], 2022**									
		Random forest	—		*0.39-0.61*		—		—	
		K-nearest neighbor	—		0.28-0.61		—		—	
**Epigenomics**										
	**Fan et al [27], 2021**									
		Random forest	0.79-0.91		0.69-0.78		0.65-0.74		0.81-0.92	
		Support vector machine	0.57-0.86		0.50-0.85		0.41-0.83		0.49-0.88	
		Neural network	*0.78-0.99*		*0.75-0.97*		*0.78-0.98*		*0.49-0.95*	
	**Payne et al [39], 2020**									
		Support vector machine	*0.77-0.84*		—		—		—	
		Linear discriminant analysis	0.72		—		—		—	
	**Qi et al [1], 2020**									
		Clustering	0.49-0.97		—		—		—	
**Microbiomics**										
	**Stevens et al [24], 2021**									
		Random forest	0.66-0.90		—		—		—	
**Multiomics**										
	**Bhak et al [6], 2019**									
		Random forest	—		0.87-0.93		0.59-0.98		0.85-1	

^a^AUC: area under the curve.

^b^Machine learning methods were evaluated based on the number of genes found in pathways implicated in mood disorders.

^c^Not reported.

^d^DeepWAS: multivariate functional unit–wide association study.

^e^DeepSEA: deep learning-based sequence analyzer.

^f^Italics represent the best performing models.

^g^The only performance metrics given were partial correlation coefficients.

The GWAS of the Taiwan Biobank used 5 machine learning algorithms to build creative models incorporating SNPs and demographic information: logistic ridge regression, support vector machine, decision tree, LogitBoost, and random forest [16]. Logistic ridge regression and LogitBoost had the best performance with an AUC >0.82 and sensitivity and specificity >0.76 [16]. In the GWAS study by Takahashi et al [36], the authors aimed to decrease overfitting by decreasing the number of null variants included in the model. They compared the performance of 6 different models: smooth-threshold multivariate genetic prediction, polygenic risk scores, genomic best linear unbiased prediction, summary data–based best linear unbiased prediction, a Bayesian hierarchical model for the analysis of complex traits, and ridge regression [36]. The smooth-threshold multivariate genetic prediction had the highest prediction accuracy with a partial correlation of 0.05 and *P* value of <.005; this model also successfully reduced overfitting [36]. The study by Sekaran and Sudha [26] used 5 different machine learning algorithms to identify genetic biomarkers: Bayesian network, support vector machine, random forest, back propagation neural network, and linear discriminant analysis. The accuracy of the Bayesian network and support vector machine was >90%; the accuracy of the other algorithms was <75% [26].

The transcriptomic study by Ciobanu et al [30] combined a random forest classifier model with Weighted Gene Coexpression Network Analysis into an algorithm called fuzzy forest that identified an association between depression and the transferrin receptor gene. The fuzzy forest classifier was able to reduce the dimensionality of the transcriptomic data and allow a predictive marker of depression to be identified with a smaller sample size [30]. In a transcriptomic study using brain tissue, extreme gradient boost (XGBoost) was chosen for its feature selection and reduction characteristics and ability to rank features by importance [18]. The AUC for the best performing model was 0.72 [18]. Furthermore, XGBoost was used in the transcriptomic study by Le et al [37], and its performance was compared to 2 tree-based pipeline optimization tools (TPOTs). XGBoost produced an accuracy of 0.59, and the standard TPOT produced a similar accuracy of 0.60 [37]. The TPOT combined with a feature set selector and the ability to slice the data into smaller subsets, produced the highest prediction accuracy of 0.68 [37].

In the multiomics study by Bhak et al [6], the authors used a random forest model and feature selection to analyze blood transcriptome and methylome data; this model correctly predicted the labels for suicide attempters and nonsuicide attempters with depression and controls. Scores on the Hamilton Rating Scale for Depression-17 were also correctly predicted by a linear regression model [6]. The microbiomic study by Stevens et al [24] used a random forest method to identify gut microbiome taxa and related metabolic pathways associated with depression. The R packages ALDEx2, DADA2, and PIME (R Foundation for Statistical Computing) analyzed the DNA sequences of the microbiota in stool samples to produce exact amplicon sequence variants, identify taxa associated with those variants using a Naive Bayes classifier, and filter the results into unique amplicon sequence variant sequences [24]. This approach differentiated between individuals with depression and healthy controls, and the results were supported by multivariate analyses with a *P* value of <.001 and effect size >0.5 [24]. Machine learning predicted metabolic pathways associated with the individuals in the depression and control groups with AUCs ranging from 0.66 to 0.9 [24].

Verma et al [19] used random forest and k-nearest neighbor methods to analyze transcriptomic data and classify patients as depressed and died by suicide, depressed and did not die by suicide, and healthy controls. K-nearest neighbor stores all cases and classifies new cases based on their similarity [19]. Using random forest, the test data were classified with an accuracy of 61.11%, and the training data were classified with an accuracy of 97.56%; with k-nearest neighbor, the accuracy was 61.11% for test data and 76.6% for training data [19].

The GWAS using the top 500 SNPs to identify biological pathways associated with depression compared the performance of random forest; least absolute shrinkage and selection operator; and ReliefF, a nearest neighbors feature selection algorithm [35]. ReliefF was the best performing algorithm, likely due to its ability to detect statistical interactions, and this method identified most genes associated with biological pathways related to depression [35]. Furthermore, ReliefF was used in a transcriptomic study and was combined with different cross-validation methods [38]. The private evaporative cooling and general elastic net algorithms had the highest accuracy on the training data, but consensus nested cross-validation had the highest accuracy on the validation data as well as low overfitting [38].

In the study of microRNAs by Qi et al [1], a regularized gradient boosted method was used to classify individuals with depression and healthy controls. The models were trained with cross-validation and 2500 iterations of parameter searches [1]. The models were then retrained using the best parameters [1]. The best model achieved an AUC of 0.93 [1]. When classifying cases as normal to mild or moderate to severe, the best model achieved an AUC of 0.76 [1].

### Unsupervised Machine Learning

The study of microRNAs by Qi et al [1] used an unsupervised clustering approach to differentiate individuals with depression from healthy controls. A total of 500 iterations of a k-means clustering method were applied to the data set [1]. They obtained 2 clusters with similar sample sizes, both with an AUC >0.70 [1].

### Deep Learning

The DeepWAS study by Arloth et al [15] used a deep learning method called deep learning-based sequence analyzer to predict the function of SNPs. Of >8 million SNPs analyzed; this method predicted 40,000 regulatory SNPs based on their affinity with an FU [15]. The AUCs ranged from 0.59 to 0.66 [15]. A regularized linear regression was used to determine which SNPs were associated with depression [15].

The DNAm study by Fan et al [27] used a support vector machine, random forest, and a neural network to predict depression based on methylation of the tryptophan hydroxylase-2 gene. The neural network had the best performance with an AUC of 0.988, sensitivity of 98.3%, specificity of 95%, accuracy of 97.4%, and positive predictive value of 98.3% [27]. In addition, they found that models combining clinical variables with tryptophan hydroxylase-2 methylation performed better than models based on clinical variables or methylation alone [27].

### Critical Appraisal

The studies’ strengths and weaknesses were identified using the Joanna Briggs Institute Critical Appraisal Checklist for Analytical Cross-Sectional Studies, as shown in Table 4. Of the 15 studies, only 2 (13%), Fan et al [27] and Qi et al [1], clearly defined the criteria for inclusion in the sample. However, in all 15 studies, the individuals and setting were described in detail. A total of 47% (7/15) of the studies classified participants as experiencing depression but did not report how depression was measured or diagnosed. This may be due to the authors using data from biobanks and not having access to specific data about the participants.

The authors did not identify possible confounding factors in 11 (73%) of the 15 studies. However, it is typical that confounding is addressed when processing variables and during feature engineering, but it may not always be described as it is such a regular process. Therefore, the questions addressing confounding factors were marked “not applicable.” The study did not investigate the cause of depression or any associated diseases or disorders. Furthermore, those 11 studies did not present strategies to deal with confounding factors. The genomic outcomes were measured in a valid and reliable way in all the studies. The statistical analyses used seemed appropriate in all 15 studies.

**Table 4:** Joanna Briggs Institute Critical Appraisal Checklist for Analytical Cross-Sectional Studies.

Question	Arabnejad et al [35], 2018	Arloth et al [15], 2020	Bhak et al [6], 2019	Ciobanu et al [30], 2020	Fan et al 27], 2021	Le et al [37], 2020	Lin et al [16], 2021	Parvandeh et al [38], 2020	Payne et al [39], 2020	Qi et al [1], 2020	Qi et al [18], 2021	Sekaran and Sudha [26], 2019	Stevens et al [24], 2021	Takahashi et al [36], 2020	Verma and Shakya [19], 2022
Were the criteria for inclusion in the sample clearly defined?	Unclear	No	No	No	Yes	No	No	No	No	Yes	No	No	No	No	No
Were study individuals and setting described in detail?	Yes	Yes	Yes	Yes	Yes	No	Yes	No	Yes	Yes	Yes	Yes	Yes	Yes	Yes
Was the exposure measured in a valid and reliable way?	Yes	Unclear	Yes	Yes	Yes	Unclear	Yes	Unclear	Yes	Yes	No	No	Yes	Yes	No
Were objective, standard criteria used for measurement of the condition?	Yes	Unclear	Yes	Yes	Yes	Unclear	Yes	Unclear	Yes	Yes	No	No	Yes	Yes	No
Were confounding factors identified?	—^a^	—	—	—	Yes	—	Yes	—	—	—	Yes	—	Yes	—	—
Were strategies to deal with confounding factors stated?	—	—	—	—	Yes	—	Yes	—	—	—	Yes	—	Yes	—	—
Were the outcomes measured in a valid and reliable way?	Yes	Yes	Yes	Yes	Yes	Yes	Yes	Yes	Yes	Yes	Yes	Yes	Yes	Yes	Yes
Was appropriate statistical analysis used?	Yes	Yes	Yes	Yes	Yes	Yes	Yes	Yes	Yes	Yes	Yes	Yes	Yes	Yes	Yes

^a^Not applicable.

## Discussion

### Principal Findings

Machine learning can enable researchers to identify specific features that impact depression, allowing providers to screen for these features in a clinical setting. In this scoping review, 15 studies published in the past 5 years reported on machine learning analysis of omics data to identify individuals with depression. Owing to the diversity of the data sources and methods, there was minimal overlap in comparable study results, indicating that this field is still in exploratory stages but will provide new avenues for future prediction of which patients are at risk of developing depression.

Future studies could help with diagnosing depression using genomic data in a more reliable way, helping to mitigate the potential biases of screening interviews. However, while the genomic studies identified many genetic variants associated with depression, the lack of overlap in study results indicates low reproducibility, which could be related to the low 40% heritability of depression. It may also be associated with the heterogeneity of depression symptoms, with different genetic variants correlating with different symptoms.

Genetic variants can be helpful in diagnosing depression, but they are not generally responsive to environmental stimuli. Most of the genomics studies in this review focused on identifying SNPs that differed between individuals with depression and healthy controls. One study focused on detecting pathways associated with depression, while another used gene probes as biomarkers [26,35]. With the varied outcomes, it was difficult to compare these 2 studies to the others and determine if the results were consistent.

Transcriptomics can identify transcripts associated with depression or genes that are differentially expressed in depression. Gene expression has some responsiveness to the environment, as does DNAm. Of the 5 transcriptomics studies, 1 (20%) used brain and blood samples, while the other 4 (80%) used only blood samples, so it was expected that the results may vary. One of the studies reported downregulation of a single gene; another study reported general dysregulation of a few 100 genes, and 1 study identified DEGs and upregulation or downregulation of related pathways [18,19,30]. Another study focused on DGMs, groups of genes that are coexpressed in individuals with depression [37]. The fifth transcriptomics study emphasized the machine learning models and reported how many genes were selected by each model [38]. It would be ideal for comparison if all the studies performed a transcriptome-wide analysis and reported upregulation or downregulation of each DEG identified.

The DNAm study of tryptophan hydroxylase-2 focused on the methylation of a single gene rather than an epigenome-wide approach, effectively limiting the results to that gene [27]. Similarly, the postpartum depression DNAm study focused on only 2 specific genes, making it impossible to compare the results of the 2 studies [39]. Epigenome-wide association studies would likely be more effective in identifying differentially expressed regions associated with depression and possibly replicating work across studies [40].

Microbiomics was an interesting approach, as it did not use blood or saliva samples to sequence genetic material from the human participant [24]. Analysis of microbiomics data obtained from stool samples found differences in the composition of gut microbiota between individuals with depression and healthy individuals [24]. Stevens et al [24] identified particular taxa that were more prominent or depleted in the 2 groups. Furthermore, they focused on identifying physiological pathways involving microbiota that were associated with depression [24]. The multiomics study identified many DEGs and DMSs related to depression [6]. This may be the most insightful method because of the volume of results. However, it might be challenging to determine which results are the most significant. In addition, in many studies, only 1 type of omics data is available, so the multiomics method is not feasible.

A total of 20% (3/15) of the studies focused on identifying biological pathways. The genomics pathways study used the top 500 genes determined through feature selection and found associations with pathways that regulate neurotransmitter signaling [35]. The transcriptomics study identified pathways related to neurotransmitter reception, postsynaptic signal transmission, synaptic depression, and receptor activation, while the multi-omics study identified the Hippo signaling pathway, which is involved in cell proliferation and affects antidepressant response [6,41]. The genomics and transcriptomics studies show relatively consistent results in finding associations with pathways affecting neurotransmitters. The multiomics study found a different type of pathway, which may reflect the heterogeneity of depression and could indicate that different mechanisms can lead to depression. Future omics studies could include pathways analysis to build upon the knowledge of which biological pathways are involved in depression.

All the machine learning methods performed well based on their individual performance metrics. However, supervised methods are preferred when attempting to identify biological features related to depression because of their interpretability. Of the 15 studies, 8 (53%) reported AUCs to indicate how well the machine learning models performed, while 5 (33%)only reported accuracy; 2 (13%) reported accuracy, sensitivity, and specificity; 1 (7%) reported partial correlation coefficients; and 1 (7%) only quantified the number of genes found in pathways related to mood disorders. A review of the literature found that the most common metric used to evaluate machine learning models was accuracy followed by sensitivity and specificity [42]. However, the use of AUC as a performance metric is increasing [42]. It was difficult to compare the performance of the machine learning models in this review due to the range of performance metrics; using a standardized metric could prove more useful when choosing a model and comparing results.

There are ethical considerations related to the prediction of depression, such as the possibility of increasing insurance premiums. The protection of patient privacy, confidentiality, and trust is central to using genomics data, especially given how sensitive the data are and how they could be used to predict the risk of future conditions. Moreover, if it becomes feasible to predict depression before an individual shows symptoms, providers will need to determine the appropriate timing for treatment. They could begin treating preemptively or wait for symptoms to manifest. Furthermore, the cost of analyzing omics data should be considered. Researchers should evaluate whether omics data have a higher predictive accuracy than formal psychiatric evaluation. If not, using omics data may not be the most cost-effective way to identify individuals with depression.

### Limitations

Finally, this scoping review is not without limitations. First, many of the studies used data from biobanks, which did not provide detailed descriptions of the participants in the data sets. This makes it impossible to know the demographics and other sample characteristics. In addition, unknown sample characteristics make the generalizability of study results unclear. Moreover, some studies did not report how depression was screened or diagnosed among patients, so it is not known if validated screening measures or formal psychiatric diagnoses were used or only patient reports were used.

### Future Work

In future research, it may be helpful to focus on machine learning methods that identify features rather than those that are more geared toward prediction. Identified features can include genetic variants, DEGs, or differentially methylated regions, which would provide more relevant information that could be used to identify depression. The long-term goal of this work is to be able to use these biomarkers for a more objective diagnosis of depression.

### Nursing Implications

Nurses are in a unique position to provide mental health support to patients when they have received appropriate training and education in psychotherapy [43]. Nurses have been called the “gateway” for care because they are typically the first point of contact with the health system and are in a position to build therapeutic relationships with patients [44]. With their skills in establishing therapeutic relationships, building rapport, active listening, observing behaviors, and noticing the effects of medications, nurses serve an extremely important role in the health promotion of patients seeking mental health support [44].

In addition, machine learning–based prediction of depression will eventually become part of common nursing clinical workflow. Therefore, it is imperative that nurses bring their expertise to the creation, evaluation, and implementation of artificial intelligence approaches to depression prediction. Of note, none of the 15 studies had nurse researchers as members of their study team. Nursing involvement in the entire life cycle of artificial intelligence will positively impact the usability and usefulness of data tools in clinical practice.

### Conclusions

This scoping review describes different types of omics data and machine learning methods used to analyze these data to predict and diagnose depression. The findings indicate that the omics methods had similar performance in identifying variants, differentially methylated sites, and differences in gene expression. All machine learning methods performed well based on the metrics provided. Further research is needed in omics methods to identify more variants and differential sites and gene expression. When variants in omics data indicate the possibility of depression, it is important for clinicians, especially nurses, to assess individuals for symptoms of depression and provide a formal diagnosis and treatment if appropriate.

## Appendix

Multimedia Appendix 1 Search strategy and keywords.

Multimedia Appendix 2 Preferred Reporting Items for Systematic Reviews and Meta-Analyses Extension for Scoping Reviews (PRISMA-ScR) checklist.

## Abbreviations

AUC: area under the curve
CpG: cytosine-phosphodiester bond-guanine
DeepWAS: functional unit–wide association study
DEG: differentially expressed gene
DGM: depression gene module
DMS: differentially methylated site
DNAm: DNA methylation
dSNP: single nucleotide polymorphisms associated with a disease
FU: functional unit
GWAS: genome-wide association study
mRNA: messenger RNA
PRISMA: Preferred Reporting Items for Systematic Reviews and Meta-Analyses
PRISMA-ScR: Preferred Reporting Items for Systematic Reviews and Meta-Analyses Extension for Scoping Reviews
SNP: single nucleotide polymorphism
TPOT: tree-based pipeline optimization tool
XGBoost: extreme gradient boost
