# Supplementary material for: Identifying Depression Through Machine Learning Analysis of Omics Data: Scoping Review
Source: JMIR Nurs. 2024 Jul 19;7:e54810. doi: 10.2196/54810 (PMC11297379; doi:10.2196/54810)
Supplement: Multimedia Appendix 1 [file nursing_v7i1e54810_app1.docx]

| **Table S1** | |
| --- | --- |
| *Search Strategy and Keywords* | |
| Database | Search String |
| PubMed | (("machine learning"[MeSH Terms] OR ("machine"[All Fields] AND "learning"[All Fields]) OR "machine learning"[All Fields] OR ("deep learning"[MeSH Terms] OR ("deep"[All Fields] AND "learning"[All Fields]) OR "deep learning"[All Fields]) OR ("neural networks, computer"[MeSH Terms] OR ("neural"[All Fields] AND "networks"[All Fields] AND "computer"[All Fields]) OR "computer neural networks"[All Fields] OR ("neural"[All Fields] AND "network"[All Fields]) OR "neural network"[All Fields]))  AND ("depressed"[All Fields] OR "depression"[MeSH Terms] OR "depression"[All Fields] OR "depressions"[All Fields] OR "depression s"[All Fields] OR "depressive disorder"[MeSH Terms] OR ("depressive"[All Fields] AND "disorder"[All Fields]) OR "depressive disorder"[All Fields] OR "depressivity"[All Fields] OR "depressive"[All Fields] OR "depressively"[All Fields] OR "depressiveness"[All Fields] OR "depressives"[All Fields] OR ("depressed"[All Fields] OR "depression"[MeSH Terms] OR "depression"[All Fields] OR "depressions"[All Fields] OR "depression s"[All Fields] OR "depressive disorder"[MeSH Terms] OR ("depressive"[All Fields] AND "disorder"[All Fields]) OR "depressive disorder"[All Fields] OR "depressivity"[All Fields] OR "depressive"[All Fields] OR "depressively"[All Fields] OR "depressiveness"[All Fields] OR "depressives"[All Fields]) OR ("depressed"[All Fields] OR "depression"[MeSH Terms] OR "depression"[All Fields] OR "depressions"[All Fields] OR "depression s"[All Fields] OR "depressive disorder"[MeSH Terms] OR ("depressive"[All Fields] AND "disorder"[All Fields]) OR "depressive disorder"[All Fields] OR "depressivity"[All Fields] OR "depressive"[All Fields] OR "depressively"[All Fields] OR "depressiveness"[All Fields] OR "depressives"[All Fields]))  AND ("genom*"[All Fields] OR "gene*"[All Fields] OR "transcript*"[All Fields] OR ("dna"[MeSH Terms] OR "dna"[All Fields]) OR ("rna"[MeSH Terms] OR "rna"[All Fields]) OR "methylat*"[All Fields] OR "epigen*"[All Fields] OR "omic"[All Fields])) |
|  | AND (2017:2022[pdat]) |
|  |  |

| Database | Search String |
| --- | --- |
| CINAHL | ( depression OR depressive OR depressed ) |
|  |  |
|  | AND ( machine learning OR deep learning OR neural network ) |
|  |  |
|  | AND ( genomic OR genetic OR transcriptomic OR DNA OR RNA OR methylation OR omic OR microbiom ) |
|  |  |
|  | **Limiters** - Published Date: 20170101-20221231 |
| Scopus | ( TITLE-ABS-KEY ( depression OR depressed OR depressive )AND  TITLE-ABS-KEY ( machine  AND learning  OR  deep  AND learning  OR  neural  AND network )AND  TITLE-ABS-KEY ( genomic  OR  genome  OR  genetic  OR  gene  OR  methylation  OR  transcriptome  OR  transcriptomic  OR  epigenetic  OR  epigenomic  OR  omic ) )AND  ( LIMIT-TO ( PUBYEAR ,  2022 )  OR  LIMIT-TO ( PUBYEAR ,  2021 )  OR  LIMIT-TO ( PUBYEAR ,  2020 )  OR  LIMIT-TO ( PUBYEAR ,  2019 )  OR  LIMIT-TO ( PUBYEAR ,  2018 )  OR  LIMIT-TO ( PUBYEAR ,  2017 ) ) |
